# Thermo-responsive polymer encapsulated gold nanorods for single continuous wave laser-induced photodynamic/photothermal tumour therapy

**DOI:** 10.1186/s12951-020-00754-8

**Published:** 2021-02-08

**Authors:** Beilei Gong, Yuanbing Shen, Huiyan Li, Xiaojun Li, Xia Huan, Jihong Zhou, Yuqing Chen, Jian Wu, Wei Li

**Affiliations:** 1 Department of Respiratory Disease, The First Affiliated Hospital of Bengbu Medical College, Bengbu, 233004 China; 2 Center for Clinical Medicine of Respiratory Disease (tumor) in Anhui, Bengbu, 233004 China; 3 Department of Medical Microbiology, MOE/NHC/CAMS Key Laboratory of Medical Molecular Virology, School of Basic Medical Sciences, Fudan University, Shanghai, 200032 China; 4 Department of Thoracic Surgery, the First Affiliated Hospital of Bengbu Medical College, 233004 Bengbu, China; 5 Department of Respiratory Disease, People’s Hospital of Shannan, Shannan, 856000 Tibet China; 6 Anhui Province Key Laboratory of Translational Cancer Research, Bengbu, 233003 Anhui China; 7 Dept. of Gastroenterology & Hepatology, Zhongshan Hospital of Fudan University, Shanghai, 200032 China

**Keywords:** Gold nanorods, Thermoresponsive, Photodynamic therapy, Photothermal therapy

## Abstract

Owing to strong and tunable surface plasmon resonance (SPR) effect and good biocompatibility, gold nanoparticles have been suggested to be a versatile platform for a broad range of biomedical applications. In this study, a new nanoplatform of thermo-responsive polymer encapsulated gold nanorods incorporating indocyanine green (ICG) was designed to couple the photothermal properties of gold nanorods (AuNRs) and the photodynamic properties of ICG to enhance the photodynamic/photothermal combination therapy (PDT/PTT). In addition to the significantly increased payload and enhancing photostability of ICG, the polymer shell in the nanoplatform also has thermo-responsive characteristics that can control the release of drugs at tumour sites upon the laser irradiation. On the basis of these improvements, the nanoplatform strongly increased drug aggregation at the tumour site and improved the photothermal/photodynamic therapeutic efficacy. These results suggest that this nanoplatform would be a great potential system for tumour imaging and antitumour therapy.
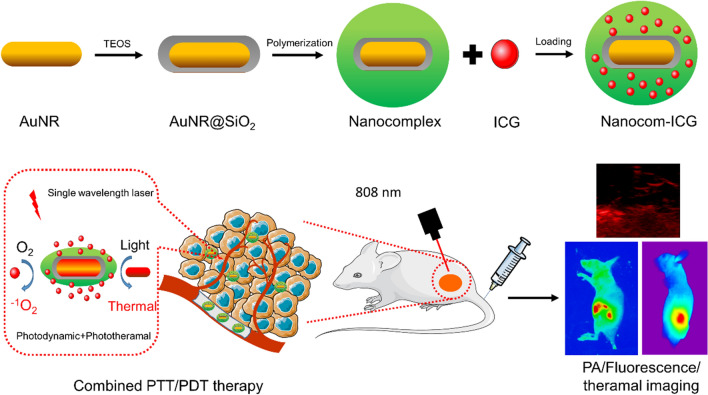

## Background

Photodynamic therapy (PDT) is a photobased therapeutic modality with three essential components (light, photosensitizer and oxygen) for the killing of malignant cells by producing highly reactive oxygen species, especially singlet oxygen (^1^O_2_), when exposed to light of a suitable wavelength. [1–4] The efficacy of PDT depends almost entirely on the generation of ^1^O_2_ created by photosensitizers after light absorption and the subsequent transfer of the excited state energy to oxygen molecules. However, most photosensitizers have intrinsic drawbacks, such as easy photodegradation, rapid blood clearance and thermal instability in aqueous solutions [5, 6]. To overcome these intrinsic drawbacks of photosensitizers, various gold nanoplatform-based nanoparticles have been developed to enhance the tumour targeting and therapeutic efficiency of the photosensitizer [7, 8].

Over the past decade, many different types of gold nanoparticles have been reported, such as gold nanorods, gold nanostars and gold nanotriangles [9, 10]. Gold nanoparticles are able to generate sufficient heat to raise the local temperature and kill cancer cells when exposed to NIR light [11, 12]. The good biocompatibility of gold nanoparticles has been demonstrated in a number of cytotoxicity studies; clinical biocompatibility studies in vivo have also shown favourable results [13–15]. Recently, Photodynamic/Photothermal therapy (PDT/PTT) combination therapy has been developed as a hopeful joint therapeutic strategy to obtain a high therapeutic index [16–18]. Photosensitizers have been intensively studied for in vitro/in vivo fluorescence imaging and have shown great potential in PDT/PTT combination therapy when incorporated into gold nanoparticles [19–21]. Therefore, combination therapy has been considered a promising strategy to improve the therapeutic efficiency and minimize side effects. However, it should be noted that previous works tended to load photosensitizers directly onto the gold nanoparticles by either physical absorption or chemical bonding between the two elements [22, 23]. Therefore, a portion of the drug cannot reach the required treatment site, which reduces the therapeutic effect. Additionally, the fluorescent photostability is markedly quenched when the drug is bound to the surface of the nanoparticle due to fluorescence resonance energy transfer (FRET) [24, 25]. Drug release from nanoparticles is induced only when stimuli are applied, including temperature, pH, light and ultrasound, thus preserving the bioactivity of the loaded drug and minimizing cytotoxic effects [26–29]. Therefore, it is promising for research workers to design a nanoprobe with high drug loading that can release the drug to the targeted tumours.

In this study, we fabricated a nanocomposite (Nanocom), which is composed of mesoporous silica coated gold nanorods with a thermo- and pH-responsive polymer shell, poly (*N*-isopropylacrylamide-co-acrylic acid). Then, we explored its combination efficacy based on the PTT effects of AuNRs and the PDT effects of ICG. Because of the external chemical properties, the drug loading of Nanocom can reach 15 wt%. In addition, the external chemical layer can cause a thermal degradation reaction and thus quickly release the loaded drug after providing 808 nm laser irradiation. In the experiment, the wavelength from the loaded photosensitive drug is the same as the surface plasmon resonance of the as-prepared AuNRs, which apparently improves the singlet oxygen production of the photosensitive drug and leads to an ideal therapeutic effect *in vitro*. Due to the protection of the loaded drug from the nanocomplex and the thermo-responsive targeted release of the loaded drug into the tumour location, when single wavelength irradiation was applied, we obtained satisfying antitumour treatment in vivo based on photodynamic and photothermal therapy (PDT/PTT). The experimental results have shown that combination therapy is a quite effective strategy for antitumour treatment.

## Materials and methods

### Chemicals and materials

Chloroauric acid (HAuCl_4_·3H_2_O), sodium borohydride (NaBH_4_), cetyltrimethyl ammonium bromide (CTAB), silver nitrate (AgNO_3_), tetraethylorthsilicate (TEOS), 3-(Methacryloxy) propyl triethoxysilane (MPS), N-isopropylacrylamide (NIPAM), N,N’-Methylenebis (acrylamide) (MBA), sodium dodecyl sulfate (SDS), potassium persulfate (KPS), poly(vinylpyrrolidone) (PVP, 24,000) were purchased from Sigma-Aldrich. Chlorine E6 (ICG) was purchased from MedKoo Biosciences. The Cell Counting Kit-8 (CCK-8) was purchased from Dojindo Laboratories. Deionized water (Millipore Milli-Q grade) with resistivity of 18.2 MΩ was used in all the experiments.

### Preparation of mesoporous silica coated gold nanorods (AuNRs)

In this experiment, gold nanorods (AuNRs) were synthesized according to the seed-mediated template-assistedprotocol [30]. Mesoporous silica coating on AuNRs was carried out according to the method of Gorelikov and Matsuurra with some medications [31, 32]. First, 4 mL of a 25 mg/mL PVP aqueous solution was added to 20 mL of the AuNR solution under gentle stirring for 15 h. The mixture was centrifuged once at 8500 rpm for 6 min, and the precipitate was dispersed in 10 mL of deionized water. Then, 100 µL of 0.1 M NaOH solution was added under stirring. Following this step, three 15 µL injections of 20% TEOS in methanol were added under gentle stirring at 30 min intervals. Finally, the mixture reacted for 3 days at 28 °C.

### Preparation of the nanocomposite

First, 200 µL of methanol containing MPS (2.5 µL) is added to 20 mL Au@SiO_2_, the mixture was stirred for 12 h and resuspend by centrifugation in 10 mL of water. Then, 10 mL of the as-prepared solution of MPS-modified Au@SiO_2_ was centrifuged at 5000 rpm for 5 min and washed twice with water and ethanol. The precipitate was dispersed in 2.5 mL of deionized water. In a typical procedure, an aqueous solution of 4.5 mL of 100 mM NIPAM, 0.5 mL of 100 mM acrylic acid, 0.8 mL of 50 mM MBA, 0.1 mL of 100 mM SDS and the MPS-modified Au@SiO_2_ solution was added to a flask. The mixture was heated to 70 °C and bubbled with nitrogen for 30 min to remove residual oxygen. Then, 1 mL of 10 mM KPS solution was rapidly added; the polymerization was allowed to proceed for an additional 4 h at 70 °C. Finally, the nanocomposites were centrifuged at 5000 rpm for 5 min and washed twice with water and ethanol to remove the unreacted monomers.

### Characterization of Nanocom

The morphology of the Nanocom was characterized by using transmission electron microscopy, operating at an accelerating voltage of 120 KV (JEOL, Japan). UV-vis spectra were measured with a Shimadzu UV-2450 UV-visible spectrophotometer. The dynamic light scattering (DLS) and zeta potential of nanoparticles was measured with Zeta potential measurements in DI water (PSS Nicomp, Santa Barbara, CA, USA).

### Drug loading and in vitro release

ICG was loaded onto the nanocomposite by mixing 0.02 mL of a 1 mg/mL ICG solution in PBS buffer with 1 mL of a 1 mg/mL nanocomposite aqueous solution for 12 h at room temperature. Then, the mixture was centrifuged at 10,000 rpm for 10 min, and the precipitate (ICG-loaded nanocomposite) was collected. The ICG concentration in the supernatant was determined by a UV-vis spectrophotometer at 785 nm to calculate the drug loading content and entrapment efficiency (loading content = weight of drug in Nanocom-ICG/weight of Nanocom-ICG; Entrapment efficiency = weight of drug in Nanocom-ICG/initial weight of drug). One millilitre of Nanocom-ICG solution was gently agitated at a series of temperatures (20 °C, 30 °C, 40 °C, 50 °C, 60 °C) for 5 min; then, the solution was centrifuged, and the supernatant was collected. The amount of released drug was imaged to compare at different temperatures using an in vivo imaging system (710 nm/790 nm as the excitation/emission wavelengths, respectively). Finally, the amount of released drug in the supernatant was determined by UV-vis spectroscopy.

### Cellular cytotoxicity assessment

The phototoxicity of Nanocom-ICG was determined in A549 cells by the CCK-8 assay. A549 cells were plated in triplicate at a density of 2000 cells per well on 96-well microplates. After 24 h of incubation, the medium was replaced with fresh medium. ICG (0–40 µg/mL), Nanocom or Nanocom-ICG (0–40 µg/mL ICG equivalents) were added to the medium and incubated overnight at 37 °C for 24, 48 and 72 h, respectively. And then, the proportion of viable cells was evaluated by a standard CCK-8 method (Dojindo).

### Cellular uptake

A549 cells were plated in triplicate at a density of 5000 cells per well on 12-well microplates. After 24 h of incubation, the medium was changed, and the cells were incubated with complete medium containing ICG (20 µg/mL ICG) or Nanocom-ICG (20 µg/mL ICG equivalent). Twelve hours later, the medium was discarded and the cells were washed three times with PBS to remove the unloaded nanoparticles, followed by the replacement of complete culture medium. Finally, fluorescence images of the cellular ICG were acquired with a confocal fluorescence microscope (Leica SP8 STED 3X, Germany) with an excitation wavelength of 633 nm. Additionally, flow cytometry (FCM) experiments were conducted to qualitatively measure cellular drug uptake. For TEM imaging of cells, the A549 cells were incubated with Nanocom-ICG (20 µg/mL ICG equivalent) at 37 °C for 24 h, and the cells were collected by centrifugation (5 min, 1200 × g), then immediately at 4 °C in 2.5% glutaraldehyde for 24 h. finally, the cell sample were prepared for TEM according to standard procedures and then observed under the TEM .

### In vitro photothermal therapy effects

To study the effects of NIR light-assisted drug release on cell death and viability, A549 cells were plated in triplicate at a density of 2 × 10^3^ cells per well on 96-well microplates. After 24 h of incubation, the medium was changed, and the cells were incubated with complete medium containing ICG (20 µg/mL ICG), Nanocom-ICG (20 µg/mL ICG equivalent) or nanocomposite at an equivalent Au elemental concentration. Twelve hours later, the medium was discarded, and the cells were gently washed with PBS. Then, complete medium was added and the cells were irradiated with an fs-laser at 808 nm ata power of 0.8 W/cm^2^ for 6 min. Then, all cells were stained with calcein AM and PI staining solution and observed by fluorescence microscopy.

### In vivo imaging

Nu/nu mice (4 weeks of age) were purchased from Anhui Slac Laboratoty Animal Co., Ltd (Anhui, China). All animals received care in compliance with the Institutional Animal Care and Use Committee of Bengbu Medical College. 1 × 10^6^ A549 cells in saline (100 µL) were subcutaneously implanted into the hind limb of nude mice. When the tumours reached a volume of approximately 100 mm^3^ (it takes about 2 weeks), the tumour-bearing mice were used in the next experiment. The nude mice with A549 tumour were injected by a lateral tail vein with saline, ICG (2 mg/kg) or Nanocom-ICG (2 mg/kg ICG equivalent), respectively. Fluorescent images were taken at 0, 1, 6, and 12 h after injection by a Maestro in vivo spectrum imaging system (Cri, Wobuen, MA, USA) (excitation: 710 nm; emission: 790 nm; integration time: 60 s). For photoacoustic (PA) imaging, the tumour-bearing mice were injected with Nanocom-ICG (2 mg/kg ICG equivalent) through the tail vein. The PA images of the tumour were acquired by using a Fujifilm Visual/VEVO LAZR-X (USA) Sonics imaging system at different timepoints (0, 1, 6, and 12 h).

### Synergistic photothermal/photodynamic therapy of Nanocom-ICG with laser irradiation

1 × 10^6^ A549 cells in saline (100 µL) were subcutaneously implanted into the hind limb of nude mice. When the tumours reached a volume of approximately 100 mm^3^ (It takes about 2 weeks), the tumor-bearing mice were anaesthetized and given a systemic injection of 200 µLof PBS, ICG (20 µg/mL), Nanocomposite or Nanocom-ICG (20 µg/mL ICG equivalent), respectively. 12 hours after tail vein injection, the PBS, Nanocomposite and Nanocom-ICG treated groups were irradiated by an 808 nm continuous wave laser for 3 min (repeat irradiation after 2 minutes). The laser treatment was carried out at a power density of 0.8 W/cm^2^. The tumours of mice were not burnt during the laser irradiation process. Additionally, the temperatures in all groups quickly declined to the normal body temperature within 2 min after completion of laser irradiation, suggesting that the treatment is likely to be safe. The temperature change in the tumour region under laser irradiation was monitored by an IR camera. Tumour growth was monitored for the following 30 days, and the tumour volumes were measured with a digital Vernier calliper. Total proteins were harvested from the tumour tissues of saline and Nanocom-ICG-treated mice after laser irradiation, and HSP70 and HSP90 were analysed by Western blot. Finally, the treated mice were sacrificed, and the corresponding tumour slices were collected for TUNEL and H&E staining according to the manufacturer’s instructions.

### Statistical analysis

All data were presented as mean ± standard deviation (SD), Statistical significance was determined by using a two-tailed student′s t-test. *P < 0.05 was regarded as statistically significant.

## Results and discussion

### Synthesis, characterization and properties of the Nanocom-ICG

**Fig. 1:**
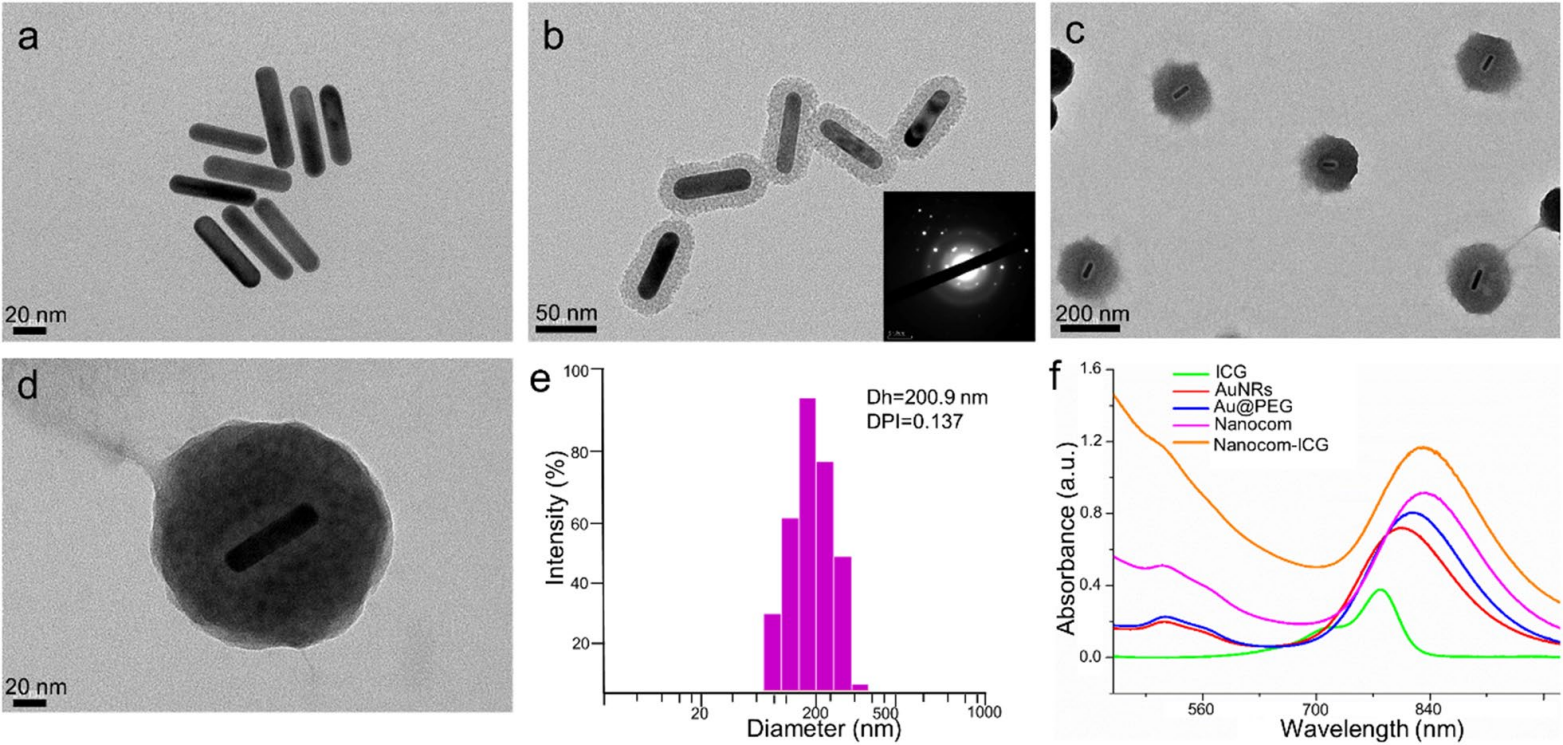
Characteristics of Nanocom-ICG. **a** TEM images of AuNRs; **b** TEM images of AuNRs@SiO_2_, insert: electron diffraction images of AuNRs@SiO_2_; **c** TEM images of Nanocom; **d** TEM images of Nanocom-ICG; **e** Hydrodynamic diameter distribution of Nanocom-ICG, And **f** UV-vis spectra of ICG, nanocom and Nanocom-ICG

In a typical experiment, AuNRs were synthesized according to the seed-mediated template-assisted protocol with some modifications. As Fig. 1a and Additional file 1: Fig. S1a show, AuNRs exhibit good uniformity with a length of 59 ± 3.6 nm and a width of 9.3 ± 1.2 nm. For the coat of silicon dioxide, the thickness of the silica layer can be tuned by adjusting the amount of TEOS in the reaction. In our study, the thickness of the coated silica layer was 15 nm (Fig. 1b and Additional file 1: Fig. S1b). A polymer layer consisting mainly of poly (*N*-isopropylacrylamide, PNIPAM) was coated onto the surface of the AuNR@SiO_2_ according to the seeded precipitation polymerization method with some modifications. The PNIPAM layer undergoes a reversible phase transition in aqueous solution from an extended hydrophilic chain to a condensed hydrophobic globule when the temperature is raised above 32 °C. As shown in Fig. 1c, d, the thickness of the PNIPAM layer was optimized to be ~ 65 nm for efficient drug loading and tumour targeting, and it is also seen from the TEM image that the prepared nanoprobe has excellent uniformity. Dynamic light scattering (DLS) further revealed that Nanocom-ICG has a narrow size distribution with an average diameter of 200.9 nm (Fig. 1e). The AuNR has two adsorption bands: a weak transverse surface plasmon resonance wavelength (TSPRW) at approximately 520 nm and a strong longitudinal surface plasmon resonance wavelength (LSPRW) at approximately 790 nm (Fig. 1f). After being coated with a silica layer, the LSPRW of the AuNRs exhibits a redshift of approximately 15 nm. After PNIPAM layer growth, the longitudinal band of gold nanorods showed a blueshift of approximately 20 nm. Obviously, the LSPRW after polymer coating and ICG loading remained in the NIR region, which permits the photos to penetrate biological tissues with relatively high transmissivity.

### Drug loading and controlled release

**Fig. 2:**
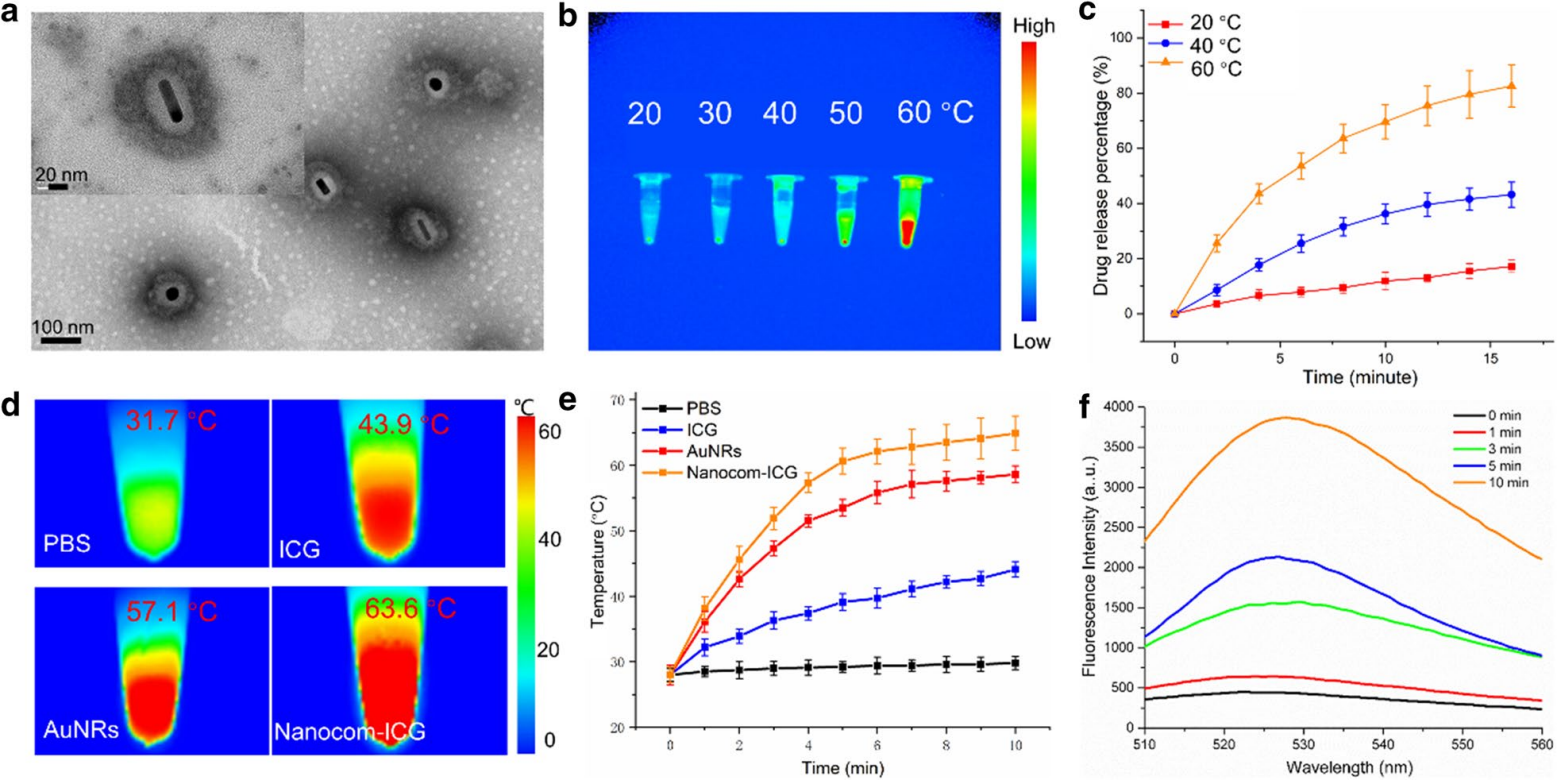
Characteristics of Nanocom-ICG. **a** TEM images of Nanocom-ICG after 5 min of laser irradiation (808 nm); **b** Fluorescence images of Nanocom-ICG incubated for 5 min at different temperatures (excitation = 710 nm); **c** Time course of ICG release from Nanocom-ICG at different temperatures; **d** NIR-thermal imaging images of PBS, free ICG (20 µg/mL), AuNRs and Nanocom-ICG (20 µg/mL ICG equivalent) after 5 min of laser irradiation (808 nm, 0.8 W/cm^2^), the volume of each group is 200 µL; **e** Temperature change from the photothermal effects of PBS, ICG, AuNRs and Nanocom-ICG; **f** SOSG fluorescence spectra of Nanocom-ICG after laser irradiation for different times (0.8 W/cm^2^), *a.u.* fluorescence unit, Ex/Em = 495/526 nm

In this work, ICG was loaded into the nanocomplex through electrostatic interactions, preparing Nanocom-ICG. The amount of ICG loaded into nanoplatform was determined by a UV/vis spectrophotometer at 780 nm, and the loading content of ICG was estimated to be 15 wt %. In aqueous solution, ICG tends to form more stable aggregates at high concentrations (> 2 µg/mL), and this is associated with a decrease in fluorescence due to the formation of fluorescence quenching centres. A decrease in peak fluorescence intensity due to the quenching effects was observed (Additional file 1: Fig. S2), which suggests that ICG forms aggregates upon adsorption into the nanocomplex. In addition to this PNIPAM layer with a surprising amount of drug loading, Nanocom-ICG has a thermal response characteristic that shrinks after the temperature increases, so that controlled release of the drug can be achieved. As shown in Fig. 2a, after Nanocom-ICG was irradiated with an 808 nm laser for 5 min, the PNIPAM layer of Nanocom-ICG was significantly shrank, and this property would be beneficial for the thermo responsive release of the drug. In aqueous solution, ICG tends to form aggregates at high concentrations, and this is associated with a decline in monomer fluorescence. When these aggregated drugs are released from Nanocom-ICG, the fluorescence can be observed due to the formation of the monomer. As expected, as the temperature of the solution increased, the fluorescence signal of ICG gradually increased (Fig. 2b). The release profile of the drug at different temperatures is shown in Fig. 2c. Nanocom-ICG shows rapid drug release when the solution temperature reaches 60 °C, and the release rate is significantly higher than in the 20 °C and 40 °C incubation groups.The drug release rate can be significantly enhanced by laser irradiation due to the heat generated by the photothermal effect of the gold nanorods, which induces shrinkage of the PNIPAM layer. These drug release results indicated that Nanocom-ICG possesses excellent temperature-triggered drug release behaviour. Furthermore, the Nanocom-ICG was suspended in saline or DMEM with 10% FBS for 2 weeks (Additional file 1: Fig. S3), and no observable agglomeration as well as the color change occurred under two different pH environments, indicating that Nanocom-ICG excellent colloidal stability.

### The photothermal and photodynamic properties of Nanocom-ICG

Gold nanoparticles have excellent photothermal conversion efficiency, which can generate very high temperatures with a short laser irradiation time. [33] In this experiment, we used infrared thermography to detect the heat-producing ability of the Nanocom-ICG under 808 nm laser irradiation. As shown in Fig. 2d, e, the temperature of Nanocom-ICG increases the fastest during the irradiation process, reaching 63.6 °C degrees in the 6th minute after irradiation, while the ICG and AuNRs groups reached temperatures of 43.9 and 57.1 °C, respectively. The results demonstrate that Nanocom-ICG benefits from the two heat sources (ICG and AuNRs) and therefore has excellent heat production capacity. Additional file 1: Fig. S4 showed that a rapid temperature increase can be observed as Nanocom-ICG with the concentrations and irradiation time rise. In additon, the temperature changes remained invariant under three irradiation/cooling cycles within 360 S demonstrating the ideal photostability of Nanocom-ICG (Additional file 1: Fig. S5). Simultaneously, the singlet oxygen (^1^O_2_) generation capacity of Nanocom-ICG was quantitatively determined by measuring the fluorescence intensity changes of a specific singlet oxygen trapper (singlet oxygen sensor green, SOSG). The fluorescence spectra are shown in Fig. 2f, and a significant increase in singlet oxygen production was observed with increasing irradiation time, indicating that Nanocom-ICG has the ability to undergo photodynamic therapy. These results demonstrate that Nanocom-ICG possessed prospective photodynamic and photothermal qualities for further combination therapy. It is also worth noting that the absorption peak of this probe overlaps with the absorption peak of ICG, and such overlap greatly increases the singlet oxygen yield of the loaded ICG by maximizing the local field enhancement and protecting the drug against photo-degradation with the help of the high absorption cross-section of the AuNRs. As expected, after 5 min of laser irradiation, the amount of ^1^O_2_ produced by Nanocom-ICG was significantly higher than that of the free ICG at the same ICG concentration (Additional file 1: Figs. S6 and S7).

### Cell viability

**Fig. 3:**
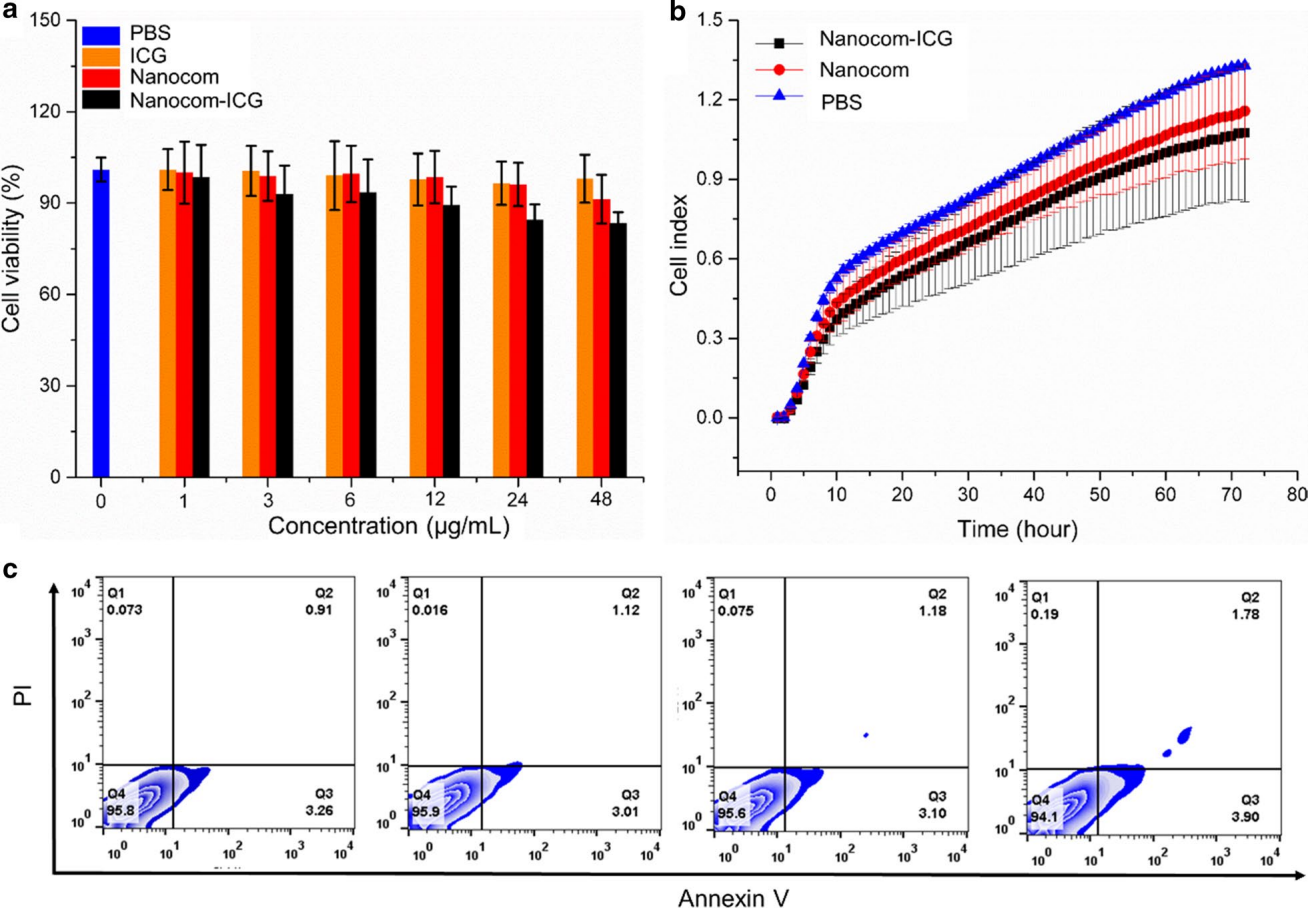
The effects of Nanocom-ICG on A549 cells. **a** The effects of PBS, ICG, Nanocom and Nanocom-ICG at different concentrations on A549 cells after 24 hours of co-incubation. **b **The cell index of A549 cells incubated with PBS and nanocom (20 µg/mL) and Nanocom-ICG (20 µg/mL ICG equivalent) for 72 h. **c** Cell apoptosis induced by Nanocom-ICG at 0, 3, 12, and 48 µg/mL ICG

To demonstrate the utility of the Nanocom-ICG for in vitro/in vivo applications, in the next experiment, the cytotoxicity of Nanocom-ICG was examined by the CCK-8 assay inA549 cells. As Fig. 3a shows, the CCK-8 data indicated no cytotoxicity of free ICG, AuNRs, nanocom or Nanocom-ICG in the concentration range of 0–24 µg/mL (ICG equivalent), and slight cytotoxicity occurred at a concentration of 48 µg/mL, even after the co-incubation time increased to 48 h or 72 h (Additional file 1: Fig. S8). Additionally, the cell index experiment results showed that nanocom and Nanocom-ICG did not affect the proliferation of cells. In addition, we used the growth index of the cells to evaluate the effect of the nanoprobe on the cells. As shown in Fig. 3b, after A549 cells were incubated with PBS, nanocom and Nanocom-ICG for 72 h, there was no significant difference in the cell proliferation curves between the three groups. Moreover, the flow analysis further proves that the nanoprobe has excellent biosafety. As shown in Fig. 3c, after A549 cells were incubated with a series of Nanocom-ICG in different concentrations (0–48 µg), there were no obvious apoptosis and necrosis in the concentration range. These results indicated that Nanocom-ICG possessed excellent biocompatibility in A549 cells. The excellent biocompatibility of the probe facilitated the next in vivo and in vitro therapeutic applications.

### Cellular uptake and localization

**Fig. 4:**
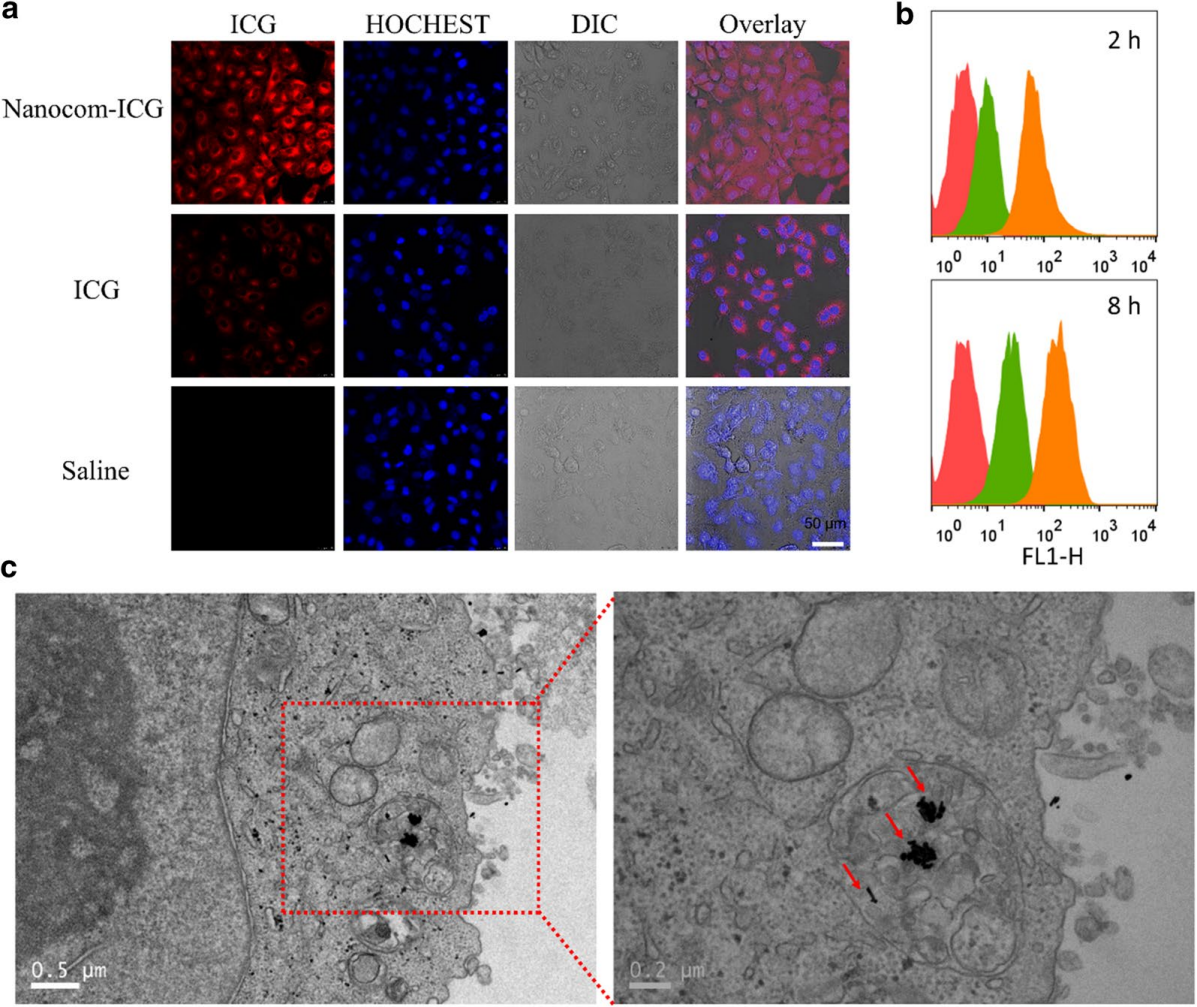
Cellular uptake. **a** Fluorescence images of ICG in A549 cells treated with saline, ICG and Nanocom-ICG (excitation = 633 nm). **b** Cellular uptake of saline, ICG and Nanocom-ICG after2 h and 8 h measured by flow cytometry. **c** TEM images of Nanocom-ICG distributed in A549 cells

In the next experiment, the fluorescence of ICG was used to assess the cellular uptake of Nanocom-ICG. The confocal fluorescence microscope showed obvious fluorescence in the cytoplasm after treatment with ICG and Nanocom-ICG, suggesting efficient cellular uptake and intracellular distribution of Nanocom-ICG (Fig. 4a). Additionally, the fluorescence intensity in Nanocom-ICG-treated cells were higher than that in ICG-treated cells, which demonstrates the prominent delivery and protection of ICG from Nanocom-ICG. In addition, we measured the cellular uptake of ICG and Nanocom-ICG by flow cytometry. The flow cytometry results show that Nanocom-ICG exhibits more efficient drug delivery capabilities after incubation with A549 cells for 2 h or 8 h than those of ICG (Fig. 4b). To more precisely observe the intracellular localization of Nanocom-ICG, the ultrastructure of the A549 cells was observed by TEM after A549 cell treatment with Nanocom-ICG for 24 h. As shown in Fig. 4c, Nanocom-ICG is clearly visible and distinct in the cell due to its high electron density, and most of the Nanocom-ICG is located in cellular vesicles, mainly endosomes and lysosomes. These observations are consistent with the results of the flow cytometry assay, and these results suggest the efficient cellular uptake of Nanocom-ICG, which is the basis for the optimal *in vitro* therapeutic effect.

### In vitro PTT/PDT therapeutic effects

**Fig. 5:**
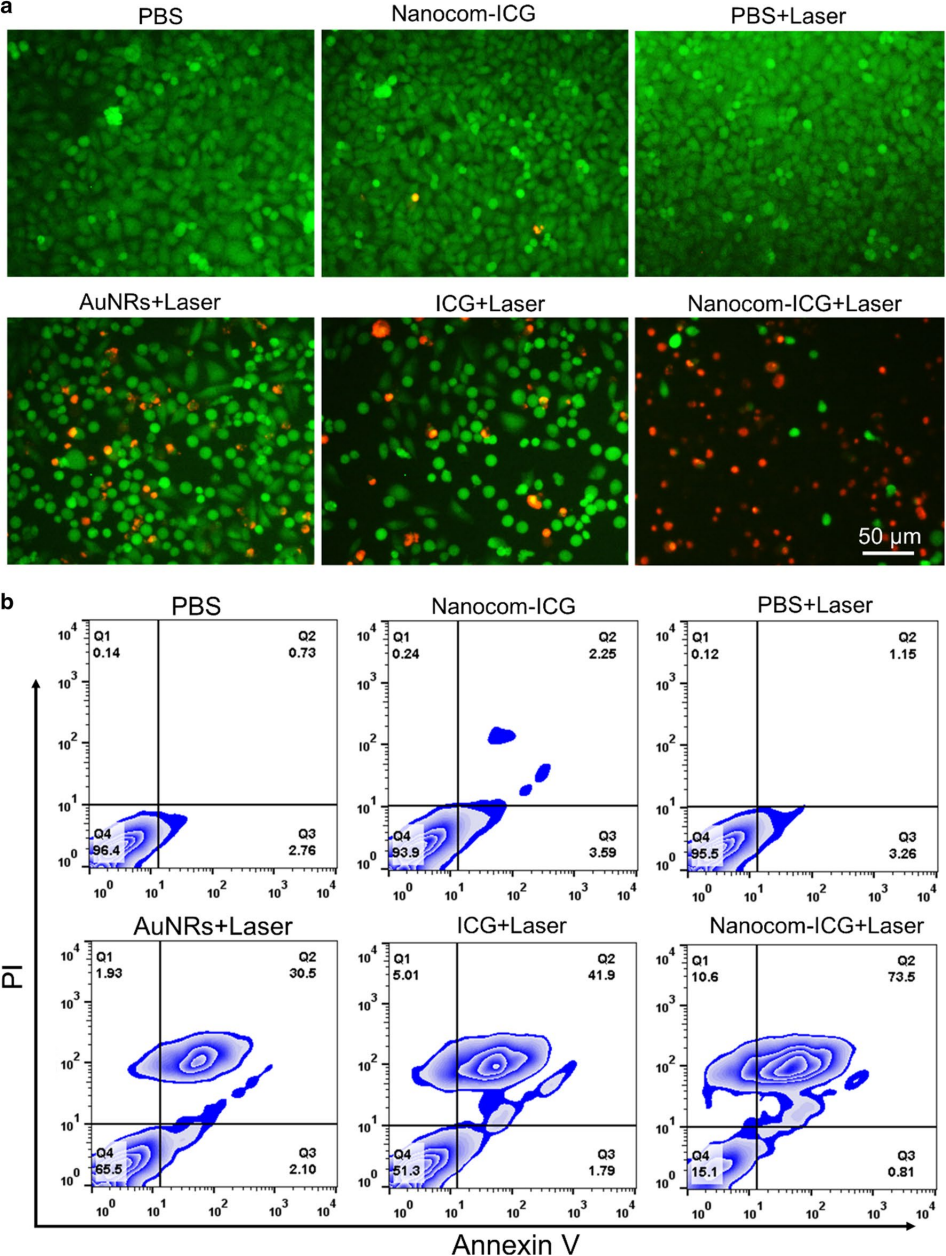
In vitro combined PTT/PDT therapy. **a** Fluorescence images of Calcein AM/PI stained A549 cells incubated with PBS, free ICG, AuNRs and Nanocom-ICG for 24 h after irradiation with an 808 nm laser (6 min, 0.8 W/cm^2^). **b** Cell viability of A 549 cells cultured with PBS, free ICG, AuNRs and Nanocom-ICG with or without 808 nm laser irradiation

In this study, the PTT/PDT combination therapy effects of Nanocom-ICG was first examined by a double-stain assay in A549 cells. A549 cells were incubated with free ICG (20 µg/mL), nanoplatform and Nanocom-ICG (20 µg/mL ICG equivalent) for 24 h and then irradiated with an 808 nm laser at a power density of 0.8 W/cm^2^ for 6 min. Finally, these cells were stained with calcein AM and PI staining solution. A series of fluorescence images are shown in Fig. 5a, and it can clearly be observed that Nanocom-ICG kills more cells than either free ICG or the nanocomplex alone after laser irradiation treatment. For the free ICG- or nanocomplex-treated groups, most of the cells were still alive after 6 min of laser irradiation. Additionally, the results of the flow cytometry apoptosis analysis were consistent with the double staining results, and as shown by the flow analysis files (Fig. 5b and Additional file 1: Fig. S9), as Nanocom-ICG induced the most cancer cell apoptosis among all treatment groups. Moreover, the cells in the PBS-treated group caused very little significant apoptosis and death of cells after irradiation, which also proved that the laser power and irradiation time we selected were safe for normal tissues. Combined with the results of cellular uptake, these results suggested that the PTT/PDT combination effect and enhanced cellular uptake are responsible for the improved therapeutic efficiency of Nanocom-ICG.

### Biodistribution of Nanocom-ICG in vivo

**Fig. 6:**
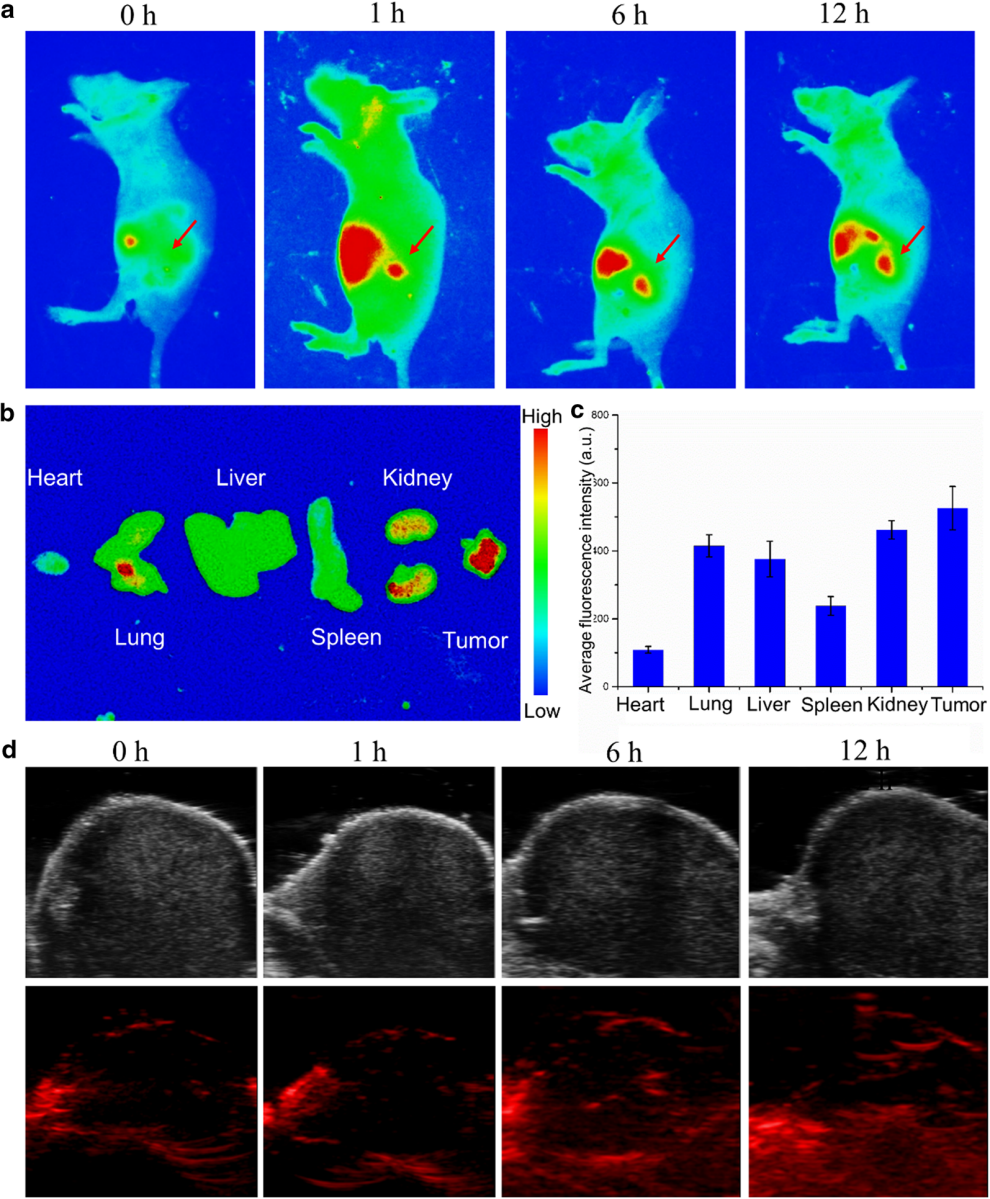
The distribution of Nanocom-ICG. **a** Real-time fluorescence imaging of A549 tumour-bearing mice before and after i.v. injection of Nanocom-ICG. The red arrows indicate the region of the tumour. **b** Ex vivo fluorescence images of mouse organs and tissue from tumor-bearing mice 12 h after injection of Nanocom-ICG. **c** Quantitative analysis of the fluorescence signals from the main organs and tumours. **d** PA images of A549 tumour-bearing mice before and after i.v. injection of Nanocom-ICG

To achieve targeted delivery and release of the loaded drug, the AuNRs were encapsulated by a PNIPAM layer. We expected that the heat generated by the photothermal effect of the gold nanorods would induce shrinkage of the PNIPAM layer. It has been shown that PNIPAM can trigger loaded drug release through photothermal effects upon irradiation with an NIR laser. In this study, Nanocom-ICG was intravenously injected into mice, and then we observed the fluorescence of ICG in the tumour-bearing mice by using a Maestro in vivo optical imaging system. As expected, a series of fluorescence images are shown in Fig. 6a. The Nanocom-ICG was mainly distributed in the liver 30 min post-injection. Over time, the fluorescence signal gradually appeared in the tumour site and reached a peak at 4 h post-injection. To further observe the distribution of photosensitive drugs in the main organs and tumour sites of experimental animals, we performed an in vivo imaging experiment. As shown in Fig. 6b, c, we can clearly observe that the fluorescence signals of the tumour site were the strongest, and these results fully indicated the ability of the nanoprobes to target the drug delivery towards the tumour site. In the following experiment, according to the photoacoustic signal of gold nanorods, we further observed aggregates of Nanocom-ICG at the tumour site by using a photoacoustic imaging system. The photoacoustic imaging images are shown in Fig. 6d. After the nanoprobe was injected intravenously into the experimental mice, the PA signals of the tumour site gradually increased and reached a peak at 12 h post-injection. These imaging results clearly demonstrated the prominent tumour-targeted aggregate and heat-induced release ability of Nanocom-ICG, which will facilitate subsequent in vivo antitumour therapy.

### Antitumour efficacy of Nanocom-ICG in vivo

**Fig. 7:**
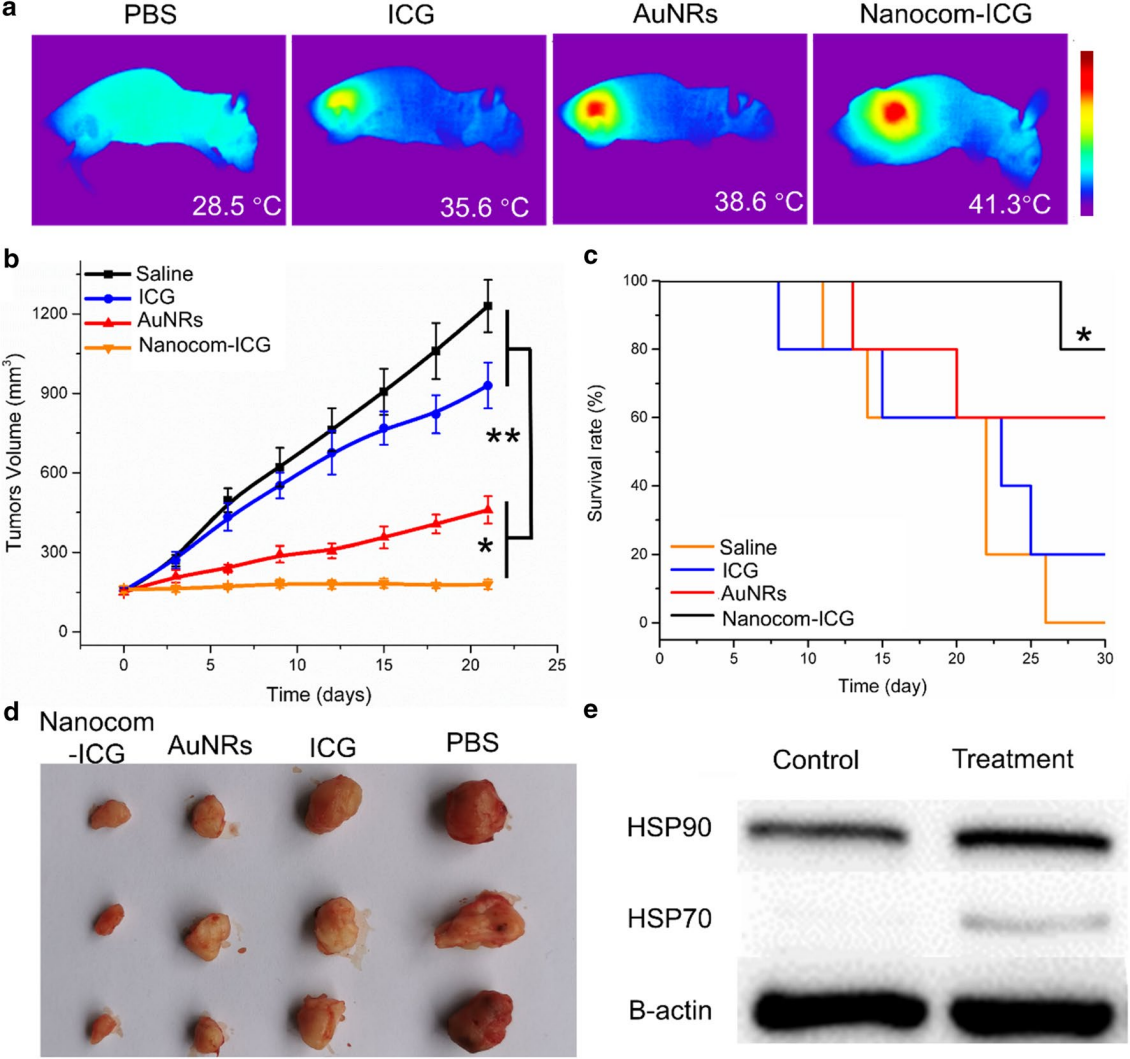
In vivo combined PTT/PDT therapy. **a** Infrared microscopic images of the A549 tumour-bearing mice after 808 nm laser irradiation (0.8 W/cm^2^, 3 min) 3 days after PBS, ICG, AuNRs and Nanocom-ICG injection. **b** Tumour growth in the various groups after laser irradiation. n = 5, *p < 0.5, **p < 0.05. **c** Survival rate in the various groups after laser irradiation. n = 5, *p < 0.5. **d** Photographs of the tumors from each groups collected 21 day after laser irradiation treatment. **e **Western blotting analysis of HSP70 and HSP90 expression in tumour tissues after laser irradiation treatment

To interpret the synergistic efficacy of PTT and PDT mediated by Nanocom-ICG, A549 tumour-bearing mice were randomly divided into 4 groups and injected with saline, ICG, Nanocom or Nanocom-ICG into a tail vein. At 4 h post-injection, the photothermal/photodynamic combination antitumour therapy was performed with 808 nm laser irradiation. In the whole process of laser treatment, the photothermal effect of Nanocom-ICG was monitored through an infrared thermal imaging camera. As shown in Fig. 7a, once the Nanocom-ICG-treated mice were exposed to the laser at a power density of 0.8 W/cm^2^, the temperature of the tumour region increased rapidly to 41.3 °C after 3 min of irradiation, and the surrounding healthy tissue showed a moderate increase in temperature. However, in the ICG- and AuNR-treated groups, the temperature of the tumour region reached 35.6 °C or 38.6 °C, respectively, after irradiation. We think that the reasons why the tumour region in the Nanocom-ICG treatment group reached the highest temperature are due to the high number of Nanocom-ICG aggregates at the tumour site and the dual heat production from the AuNRs and ICG. In addition, one minute after laser irradiation, the temperature of the tumour region in each group quickly declined back to body temperature (Additional file 1: Fig. S10), suggesting that the power density and irradiation time are likely to be safe.

**Fig. 8:**
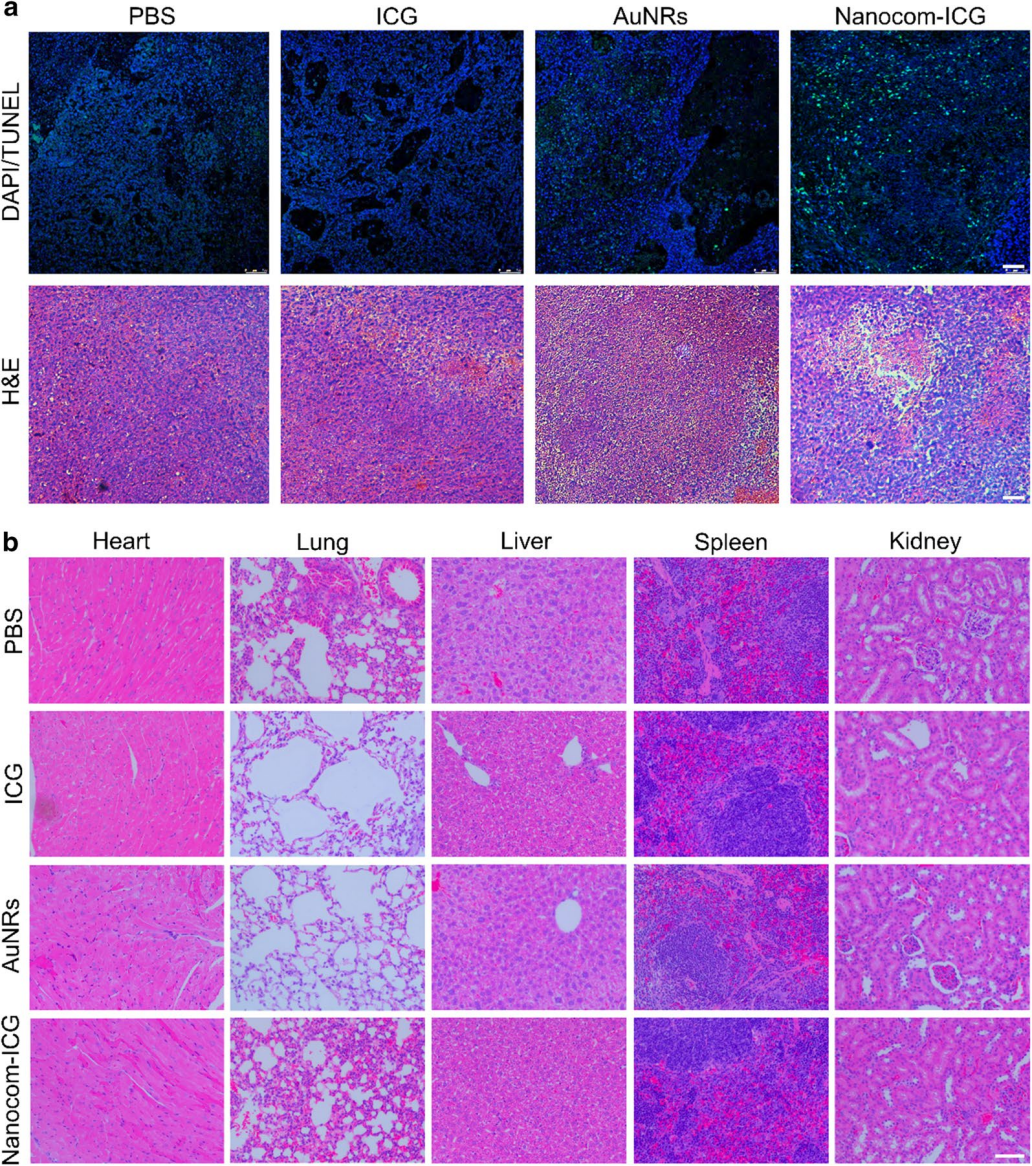
**a** TUNEL and H&E staining images of the tumours after combination therapy for 21 days; all scale bars are 100 µm. **b** Histopathological microsection of main organs from each groups collected 21 day after i.v injection of saline and Nanocom-ICG

Fourteen days after laser irradiation, as shown in Fig. 7b, in the free ICG and nanoplatform treatment groups, the tumour growth was relatively rapid. Moreover, the tumour volumes of the free ICG and nanoplatform groups were significantly larger than those of the Nanocom-ICG group, indicating that single thermal therapy or photodynamic therapy with mild laser treatment alone is ineffective in inhibiting tumour growth (Fig. 7d). In addition, consistent with the tumour growth results, the Nanocom-ICG group had the highest survival rate among all experimental groups (Fig. 7c). In addition, Western blot analysis of two main HSP members: HSP90 and HSP70, which are overexpressed in the cells under heat stress. As shown in the Fig. 7d, in the tumour tissues revealed significantly increased expression after treatment with Nanocom-ICG and laser irradiation, proving the enhanced thermal therapy effects of Nanocom-ICG. Subsequently, we performed TUNEL and H&E experiment to observe the therapeutic effects in the tumour tissue in all experimental groups. The results are shown in Fig. 8a and further confirmed that Nanocom-ICG achieved the best treatment effect. Compared with other treatment groups, many dead cells were clearly observed at the tumour site of the Nanocom-ICG treated group. The excellent antitumour therapeutic benefits from the efficient aggregates of Nanocom-ICG in the tumour region and controlled drug release resulted in photodynamic/photothermal combination therapy. In addition, there were no obvious damage or inflammatory lesions observed in the main organs (liver, lung, heart, spleen, kidney) of the Nanocom-ICG treatment group 21 days after i.v. injection (Fig. 8b), demonstrating that Nanocom-ICG does not cause obvious side effects and shows excellent biocompatibility.

## Conclusions

**Scheme 1:**
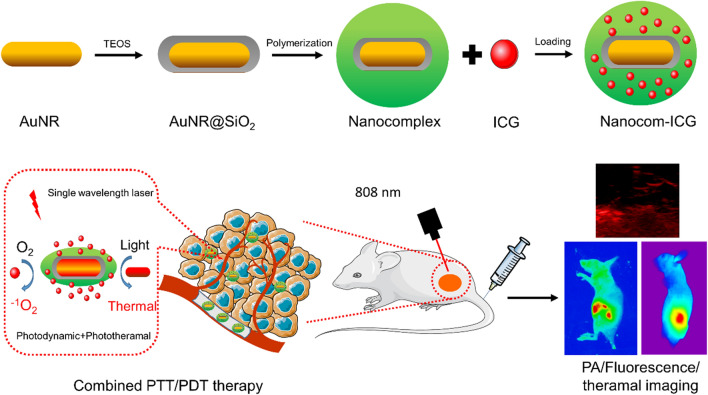
Schematic illustration of the design of Nanocom-ICG in combined PTT/PDT therapy

In summary, we have successfully designed and validated a new PTT/PDT combination therapeutic strategy for tumours by fabricating a nanoprobe composed of thermo-responsive polymer encapsulated AuNRs incorporating ICG. The thermo-responsive polymer layer strongly increased the drug loading capacity with the additional benefit of improving ICG stability by facilitating the formation of ICG J-aggregates and promoting the selective accumulation of ICG in the tumour region. Additionally, Nanocom-ICG showed minimal cytotoxicity and high biocompatibility in cellular experiments. In this study, Nanocom-ICG exhibited excellent antitumour effects both in vitro and in vivo. These results suggest that coordinated photodynamic/photothermal therapy after single NIR laser irradiation is responsible for the improved therapeutic efficiency of Nanocom-ICG. Therefore, we can conclude that further development of this combination therapy strategy opens new windows for cancer therapy (Scheme 1).

## Supplementary Information

**Additional file 1.** Additional figures.

## Data Availability

All data generated or analysed during this study are included in this publised article.

## Abbreviations

ICG: Indocyanine green
AuNRs: Gold nanorods
PDT: Photodynamic therapy
PTT: Photothermal therapy
SPR: Surface plasmon resonance
FRET: Fluorescence resonance energy transfer
CCK-8: Cell Counting Kit-8
DLS: Dynamic light scattering
ICG: Indocyanine green
