# Supplementary material for: Thermo-responsive polymer encapsulated gold nanorods for single continuous wave laser-induced photodynamic/photothermal tumour therapy
Source: J Nanobiotechnology. 2021 Feb 8;19:41. doi: 10.1186/s12951-020-00754-8 (PMC7869504; doi:10.1186/s12951-020-00754-8)
Supplement: Supplementary file 1 — Additional file 1. Additional figures. [file 12951_2020_754_MOESM1_ESM.docx]

**Thermoresponsive polymer encapsulated gold nanorods for single continuous wave laser**-**induced photodynamic/photothermal tumor therapy**

Beilei Gong, Yuanbing, Huiyan Li, Xiaojun Li, XiaHuan, Jihong Zhou, Yuqing Chen, Jian Wu, Wei Li


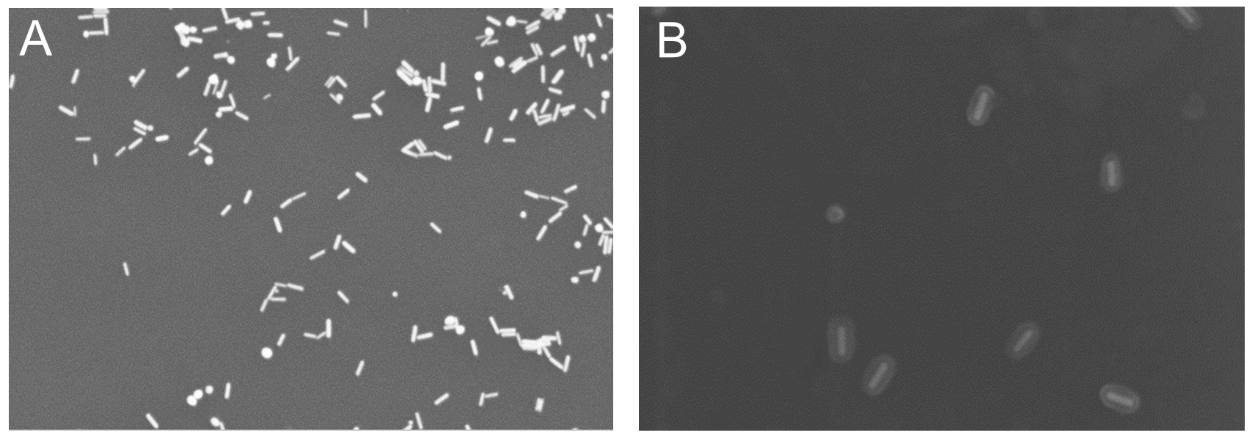


Figure S1 (A) SEM images of AuNRs, (B) SEM images of AuNRs@SiO_2_.


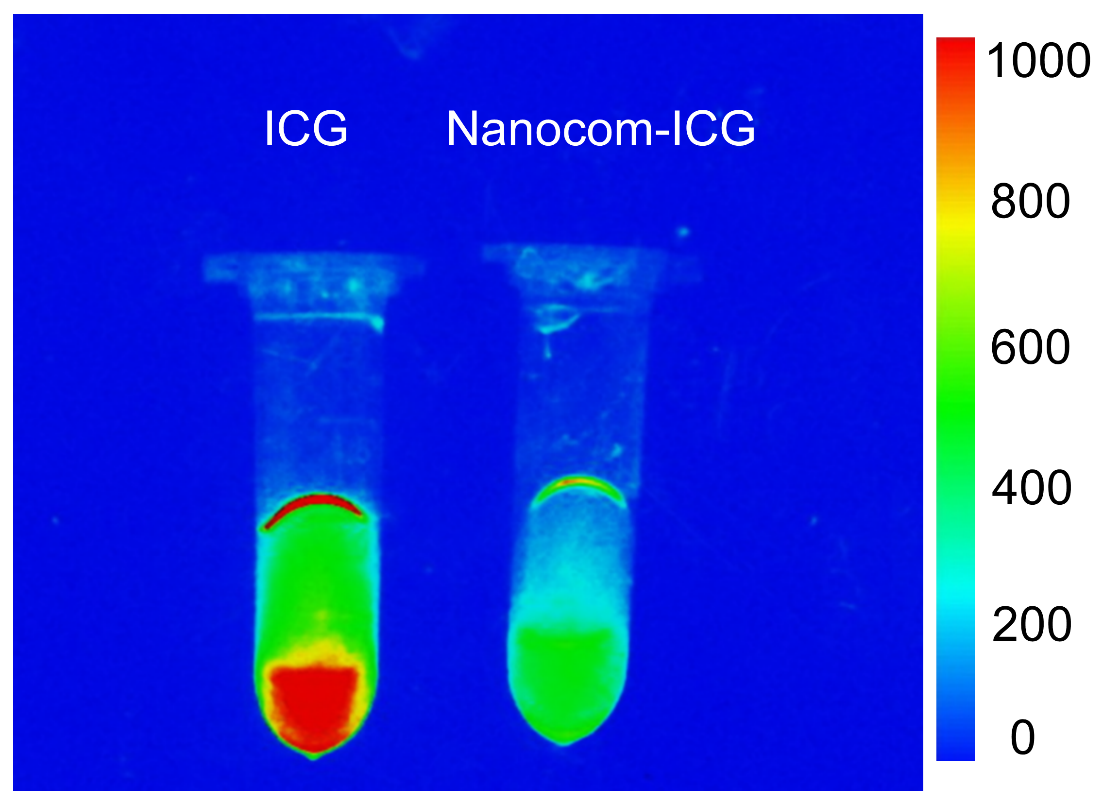


Figure S2. The fluorescence pictures of ICG and Nanocom-ICG (excitation=710 nm, emission=815 nm).


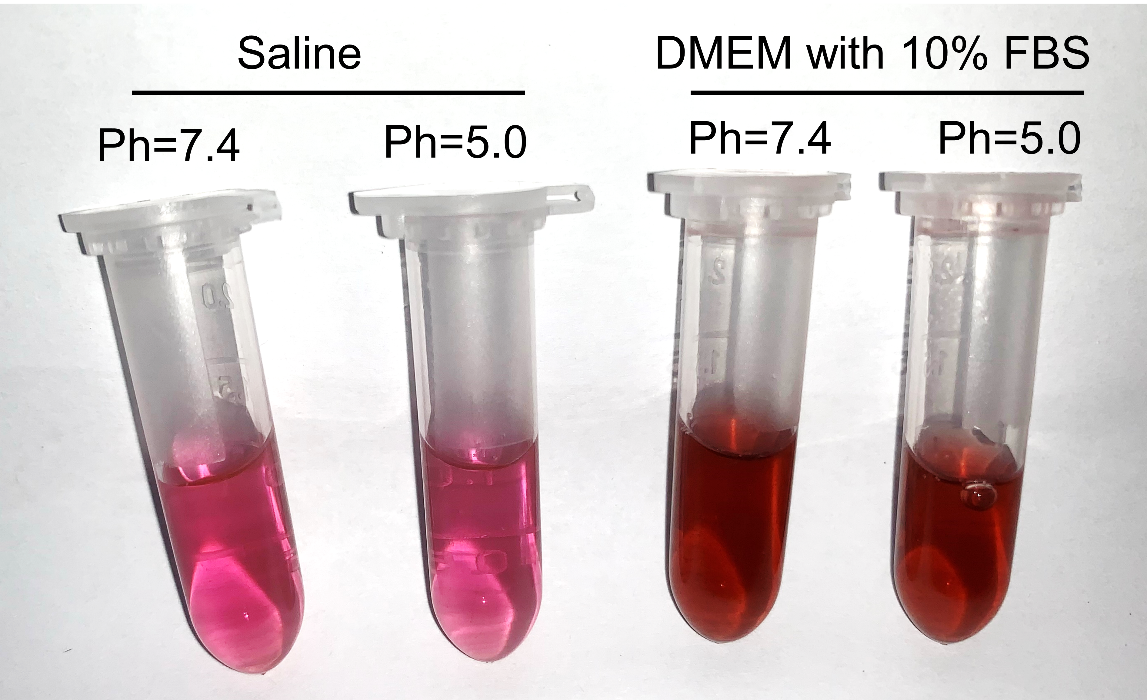


Figure S3. Digital photo of Nanocom-ICG dispered in different solutions at different pH values for 2 weeks.





Figure S4. The temperature changes of Nanocom-ICG at difference concentrations (ICG, 0–20 μg /mL) under laser irradiation for 10 min (808 nm, 0.8 W/cm^2^ ).


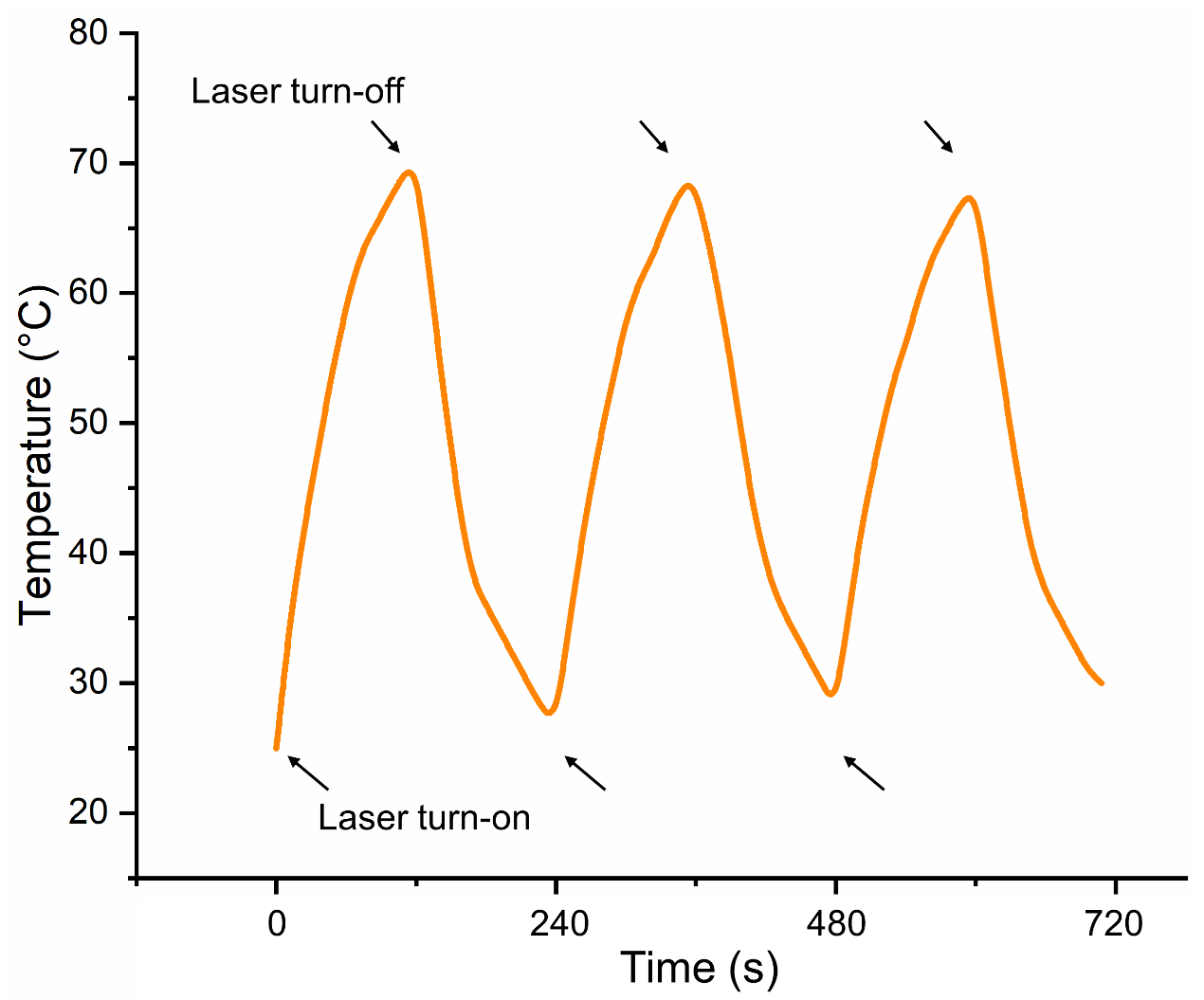


Figure S5. Temperature elevation of Nanocom-ICG (20 μg/mL ICG equivalent) under three irradiation/cooling cycles (808 nm, 0.8 W/cm^2^ ).


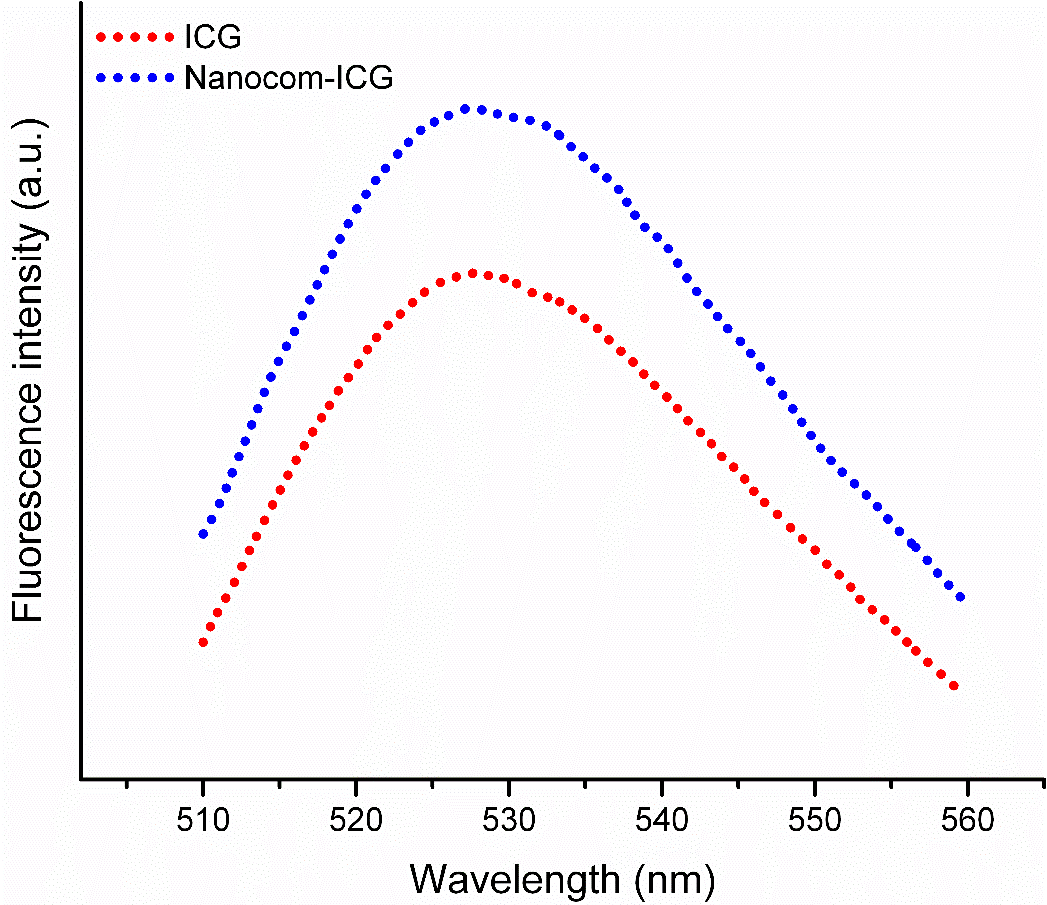


Figure S6. The SOSG fluorescence spectra of ICG and nanocom-ICG after laser irradiation for 5 minutes (0.8 W/cm^2^).


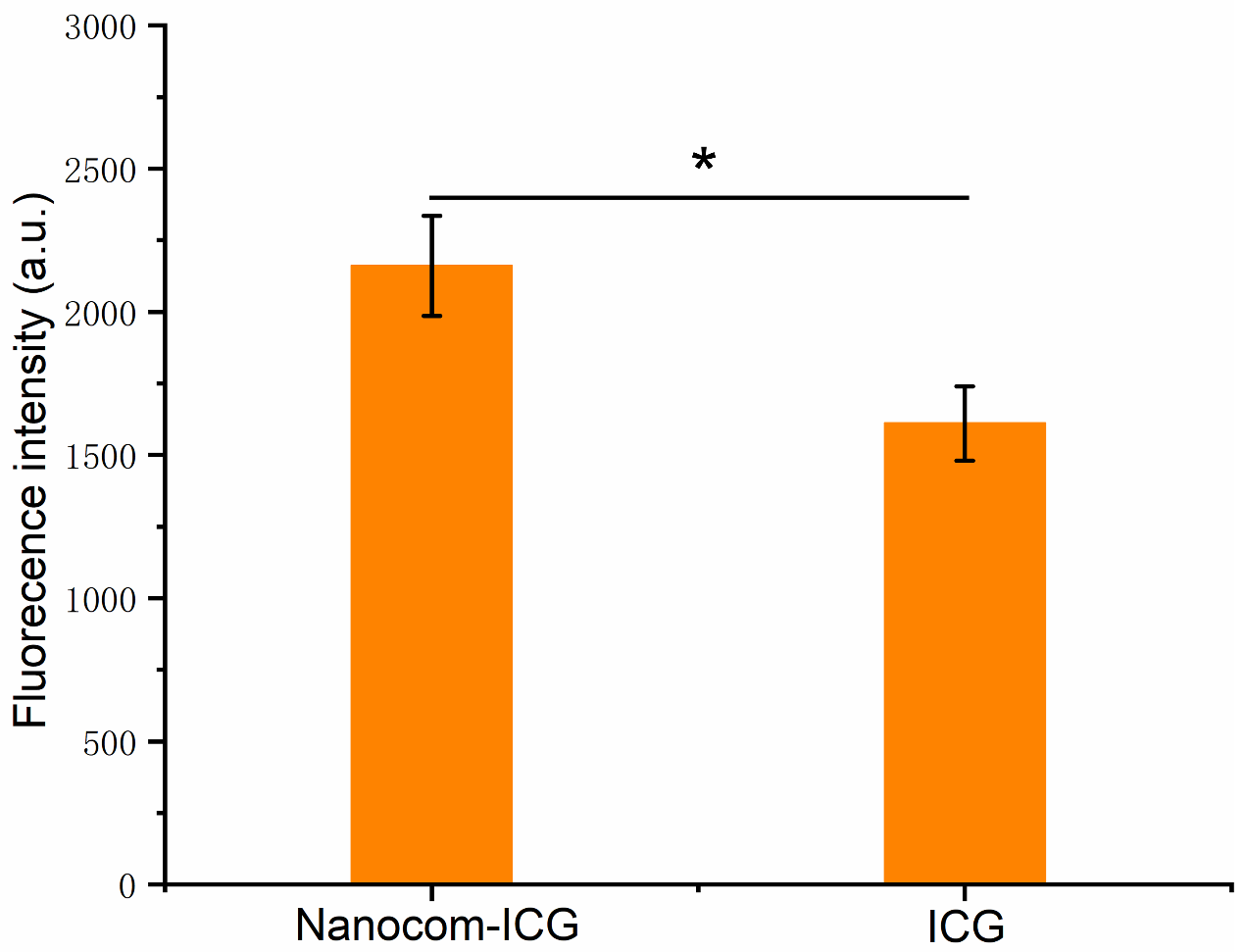


Figure S7. Statistical analysis of singlet oxygen content generated by ICG and the material after 5 minutes of laser irradiation (0.8 W/cm^2^). n = 3, **p*< 0.05.


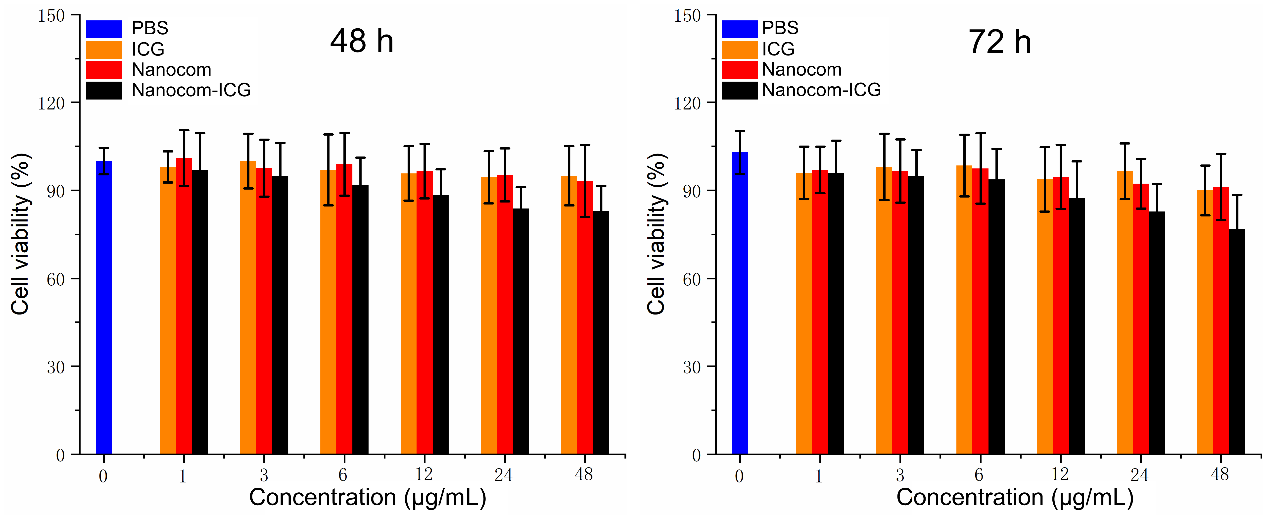


Figure S8. The effects of PBS, ICG, Nanocom and Nanocom-ICG at different concentrations on A549 cells after 48 and 72 hours of co-incubation, respectively.


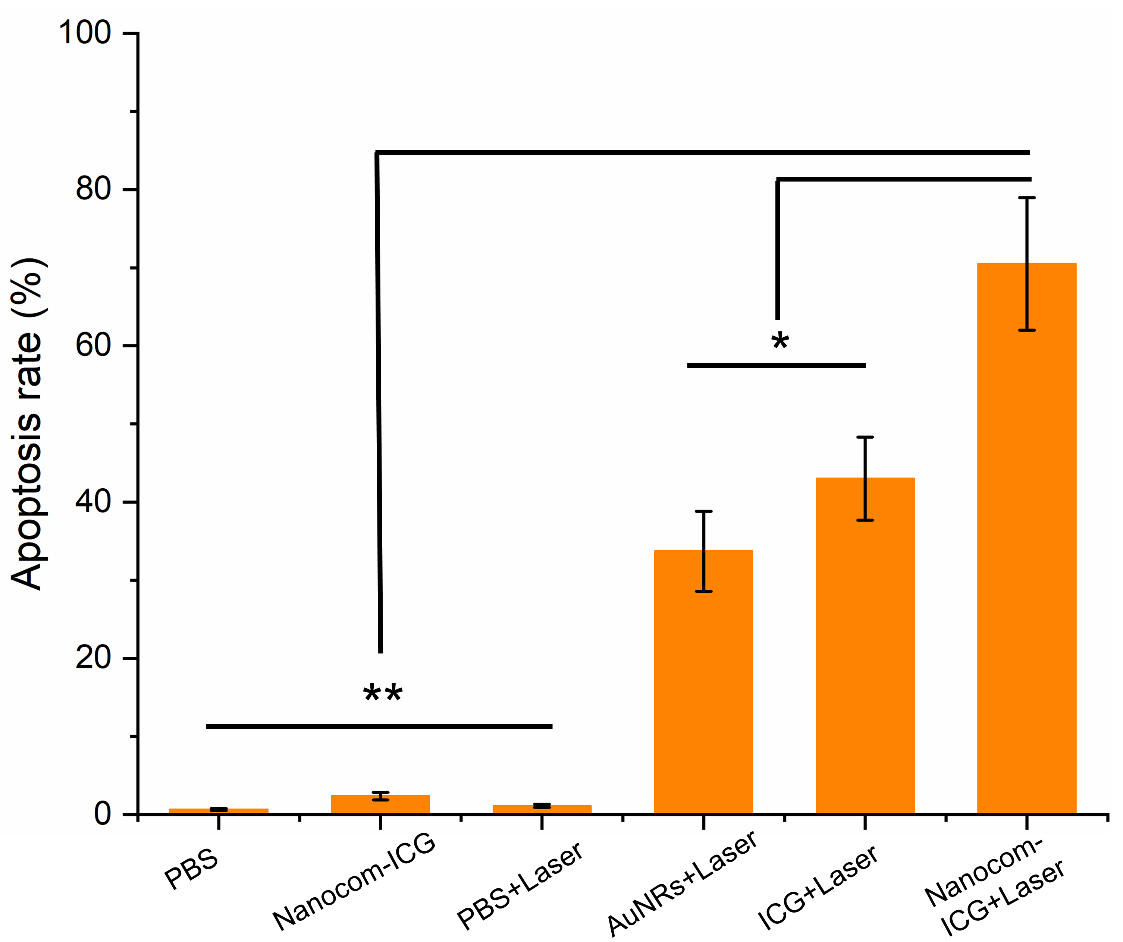


Figure S9. Relative viability of A 549 cells cultured with PBS, free ICG, AuNRs and Nanocom-ICG with or without 808 nm laser irradiation. n=5, *p<0.5, **p<0.05.


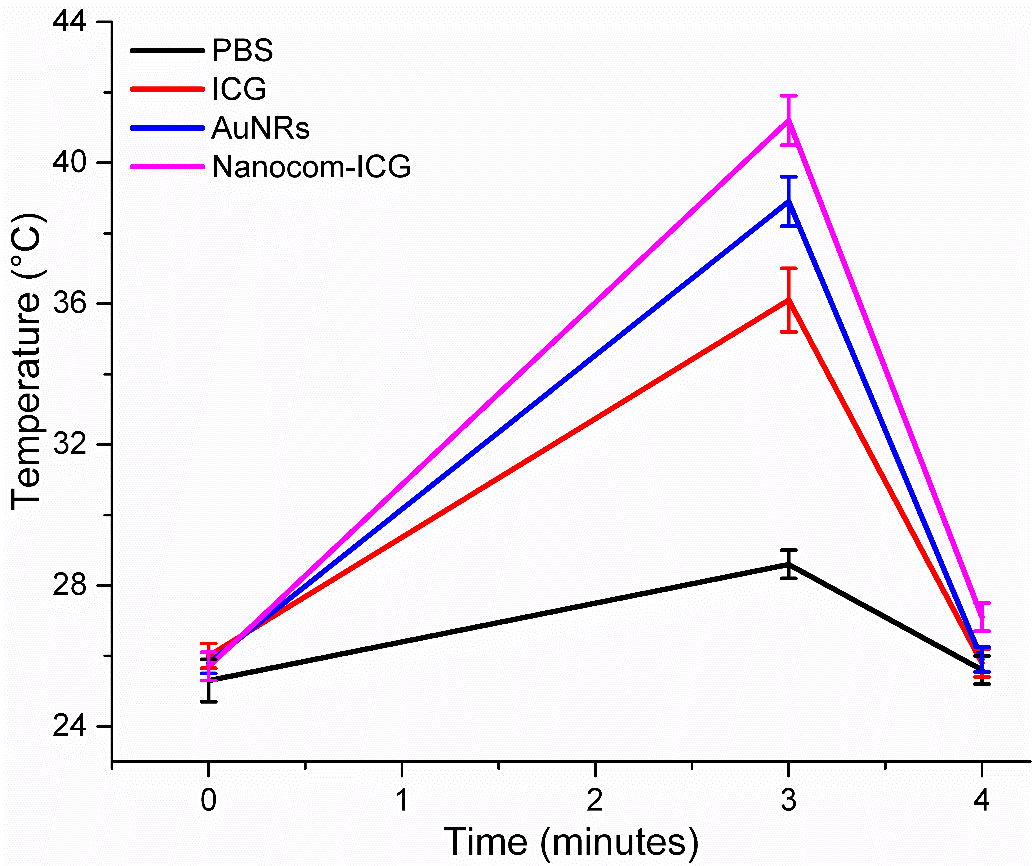


Figure S10. The temperature curves of tumor tissue regions of tumor-bearing mice during laser irradiation.
